# Alterations in Circulating Amino Acid Metabolite Ratio Associated with Arginase Activity Are Potential Indicators of Metabolic Syndrome: The Korean Genome and Epidemiology Study

**DOI:** 10.3390/nu9070740

**Published:** 2017-07-12

**Authors:** Jiyoung Moon, Oh Yoen Kim, Garam Jo, Min-Jeong Shin

**Affiliations:** 1 Department of Public Health Sciences, BK21PLUS Program in Embodiment: Health-Society Interaction, Graduate School, Korea University, Seoul 02841, Korea; answldud8503@naver.com (J.M.); grcho94@korea.ac.kr (G.J.); 2 Department of Food Science and Nutrition, Dong-A University, Busan 49315, Korea; oykim@dau.ac.kr

**Keywords:** metabolic syndrome, arginase activity, ornithine, citrulline, proline, arginine, bioavailability

## Abstract

Upregulated arginase activity, which competes with nitric oxide synthase (NOS), impairs nitric oxide production and has been implicated in various metabolic disorders. This study examined whether circulating amino acid metabolite ratios are associated with arginase and NOS activities and whether arginine bioavailability is associated with metabolic syndrome (MetS). Data related to arginase and NOS activities were collected from non-diabetic Koreans without cardiovascular disease (*n* = 1998) in the Ansan–Ansung cohorts (2005–2006). Subsequently, correlation and multivariate logistic regression analyses were performed. With the increase in the number of MetS risk factors, ratios of circulating amino acid metabolites, such as those of ornithine/citrulline, proline/citrulline, and ornithine/arginine, also significantly increased, whereas arginine bioavailability significantly decreased. These metabolite ratios and arginase bioavailability were also significantly correlated with MetS risk-related parameters, which remained significant after adjusting for covariates. In addition, logistic regression analysis revealed that high ratios of circulating metabolites and low arginine bioavailability, which indicated increased arginase activity, were significantly associated with a high MetS risk. This study demonstrated that altered ratios of circulating amino acid metabolites indicates increased arginase activity and decreased arginine bioavailability, both of which can be potential markers for MetS risk.

## 1. Introduction

Metabolic syndrome (MetS) is a continuously increasing global epidemic [1] and a key pathological condition leading to the development of type 2 diabetes (T2DM), cardiovascular disease (CVD) [2,3,4,5,6], and nonalcoholic fatty liver disease [4,6,7,8,9].

**Figure 1:**
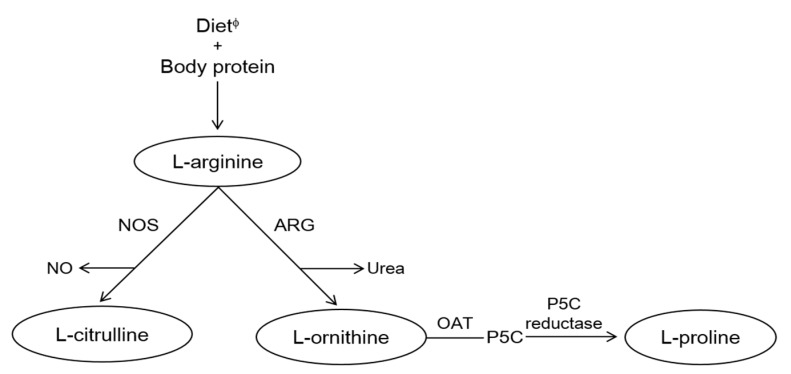
Arginine metabolism. ARG, arginase; NO, nitric oxide; NOS, nitric oxide synthase; OAT, ornithine aminotransferase; P5C, l-pyrroline-5-carboxylate. ^φ^ Major dietary sources of l-arginine are meat, poultry, fish, dairy products, nuts, etc.

Recently, arginase blockade has attracted a lot of interest as a promising therapeutic target for treating diabetes-induced vascular dysfunction [10,11,12]. Arginase inhibition has been reported to increase the availability of l-arginine to nitric oxide synthase (NOS) pathway for nitric oxide (NO) production which triggers the biological process for vasodilation by relaxing smooth muscle cells, leading to improvement of endothelial dysfunction in patients with metabolic disorders (i.e., CVD and T2DM) [10]. Our previous study has also demonstrated that arginase inhibition ameliorates obesity-induced abnormalities in hepatic lipids, endothelial function, and whole-body adiposity [11,12]. In addition, l-arginine supplementation has been reported to enhance whole-body insulin sensitivity and reduce plasma levels of fatty acids and triglycerides (TGs), indicating that it is a potentially effective treatment strategy for obesity and MetS [13,14,15,16,17]. Arginase catalyzes l-arginine hydrolysis into urea and l-ornithine, a proline precursor, and competes with NOS for l-arginine in combined NO and citrulline production; this regulates NOS activity and NO (Figure 1) [18,19]. The competitive nature of interaction between arginase and NOS indicates that increased arginase activity and/or arginase-mediated l-arginine depletion may lower NO bioavailability, which may potentially be associated with mechanisms underlying metabolic disorders, such as T2DM and cardiovascular dysfunction [20]. Thus, arginase and NOS activities may play a major role in regulating pathological/metabolic conditions by contributing to the alteration in arginine (substrate) bioavailability and ratios of secondary amino acid metabolites (namely those of ornithine, citrulline, and proline).

In this study, therefore, we hypothesized that circulating amino acid metabolite ratios may reflect arginase and NOS activities, and arginine bioavailability is associated with MetS risk, which can be useful indicators for assessing MetS risk.

## 2. Materials and Methods

### 2.1. Study Participants

This is a cross-sectional analysis of data from an ongoing prospective cohort study which is a community-based cohort study with data collected from the Korean Genome and Epidemiology Study (KoGES). Detailed information on the study design and aims of the KoGES has been reported [21]. In brief, a total of 10,030 individuals aged 40–69 years who lived in Ansan (urban) and Ansung (rural) were recruited for a baseline in 2001–2002, aiming to establish a national genomic cohort and examine the epidemiologic characteristics in Korea. All participants visited a community clinic for questionnaire-based interviews on demographic information, lifestyle, health condition, and medical history, and for anthropometry and clinical examination, and follow-up examinations were conducted biennially. The present study was based on the data on the third follow-up examination during May 2005–November 2006. Of the 7515 participants, this study was conducted with 2580 participants whose information for metabolites was available. After we excluded subjects who had diabetes and cardiovascular diseases, a total of 1998 subjects (913 men and 1085 women aged 43–74 years) were finally included for the analysis of this study. The written informed consent was obtained from each participant, and the study protocol was approved by the Institute Review Board at the Korea University (KU-IRB-16-EX-272-A-1).

### 2.2. General Information, Anthropometric and Biochemical Measurements

Survey questionnaires were administered by trained interviewers to obtain demographic and behavioral information (i.e., information on age, sex, physical activity, cigarette smoking, and alcohol consumption). Smoking status and drinking status was classified into three categories: never, former, and current. Participants were asked how long they had participated in five types of activities (sedentary, very light, light, moderate, and intense physical activity). The total metabolic equivalents (METs) were calculated by summing the METs for each type of activity (1.0 for sedentary, 1.5 for very light, 2.4 for light, 5.0 for moderate, and 7.5 for intense activities) [22]. Physical activity was divided into three categories based on the total METs: low (<20), moderate (20–40), and high (≥40) level of activities. Height and body weight (kg) were measured to the nearest 0.1 cm or 0.1 kg while wearing lightweight clothes without shoes. BMI was calculated as weight in kilograms divided by height in meters squared (kg/m^2^). Waist circumference (WC, cm) was measured at the midpoint between the lowest rib and the iliac crest in a standing position in 0.1 cm unit, and the average of three repeated measurements was used in the analysis. Repeated measurements of blood pressure (BP) were performed by a trained technician using a mercury sphygmomanometer. Two readings were taken on the left and right arms of subject in a lying position with a 5-min rest between readings. The measurements were recorded to the nearest 2 mmHg and its average was calculated for systolic and diastolic BPs (SBP, and DBP). The blood samples were collected after at least 8 h of fasting for assays of triglycerides (TG; mg/dL), high-density lipoprotein cholesterol (HDLC; mg/dL), fasting blood glucose (FBG; mg/dL), total cholesterol (TC; mg/dL), aspartate aminotransferase (AST; IU/L) and alanine aminotransferase (ALT; IU/L) measured using an automatic analyzer (ADVIA 1650 and 1680, Siemens, Tarrytown, NY, USA). Low-density lipoprotein cholesterol (LDLC; mg/dL) were calculated using the Friedwald equation [23]: LDLC (mg/dL) = TC (mg/dL) − HDLC (mg/dL) − (TG (mg/dL)/5) with TG levels below 400 mg/dL.

### 2.3. Metabolite Measurement

Targeted metabolomic measurements were performed using the Biocrates platform method using the AbsoluteIDQ^TM^ p180 Kit (BIOCRATES Life Sciences AG, Innsbruck, Austria) with stable nternal standards as reference for the calculation of all metabolite concentrations. AbsoluteIDQ^TM^ p180 Kit contains a 96-well plate with a filter plate. A total of 10 uL serum was loaded on a filter, and then extracts were analyzed by FIA-MS/MS and LC-MS/MS method. Quality assessment of the metabolite concentration measurement was performed with the MetVal^TM^ software package (BIOCRATES Life Sciences AG, Innsbruck, Austria), and quality control (QC) was performed using a calibrator and QC samples included on each plate. Finally, 139 metabolites were detected and quantification by analyzing 36 QC serum samples. For this study, four circulating amino acid metabolites including ornithine, proline, citrulline, arginine were selected and the ratios such as ornithine/citrulline, proline/citrulline, ornithine/arginine, and arginine bioavailability related to the arginase activity were tested to evaluate the association with MetS risk.

### 2.4. Definition of Metabolic Syndrome

MetS was defined by the guidelines of the National Cholesterol Education Program Adult Treatment panel III [24] with a combination of the World Health Organization and Asian Pacific cutoff value for WC [25]. Briefly, subjects who met at least three of the following criteria were considered as having MetS: (1) SBP ≥ 130/DBP ≥ 85 mmHg or antihypertensive drug use; (2) TG ≥ 150 mg/dL or TG-lowering medication use; (3) HDLC ≤ 40 mg/dL in men and ≤ 50mg/dL in women or HDLC-increasing drug use; (4) WC ≥ 90 cm in men and ≥ 80 cm in women; and (5) FBG ≥ 100 mg/dL or antidiabetic drug use (insulin or oral agents).

### 2.5. Statistical Analysis

All analyses were performed using Stata SE 12.0 (Stata Corp, College Station, TX, USA). Continuous variables measured in this study were presented as mean ± standard error (SE), and categorical variables are expressed as percentage (%). Differences between MetS people and non-MetS people were determined with Student’s *t*-tests and a general linear model (GLM) with Bonferroni’s multiple comparisons test considering potential confounding factors for continuous data and the chi-square test for the categorical data. The potential confounding factors included sex, age, BMI (log-transformed), smoking status (never, former, current), and drinking status (never, former, current). Because of skewed distribution, body weight, BMI, other parameters (TG, HDLC, FBG, AST, and ALT), and all metabolites (ornithine, citrulline, proline, arginine) were logarithmically transformed before analysis. General linear model (GLM) with Bonferroni’s multiple comparisons test were also used to compare differences in ratios of amino acid according to MetS risk status divided into three groups based on the number of MetS risk factors (0, 1–2, ≥3), after adjusting for potential confounding valuables. Pearson correlation coefficients (*r*-values) were calculated to determine the relationship between circulating amino acids metabolites and MetS risk factors or related biochemical parameters, including TG, HDLC, FBG, SBP, DBP, TC, LDLC, AST and ALT. The partial Spearman correlations were also estimated adjusting for sex, age, BMI, and smoking/drinking status. In addition, logistic regression analyses were carried out to obtain the odds ratios (ORs) and 95% confidence intervals (CIs). We evaluated the associations of circulating amino acids metabolites levels with MetS. All statistical analyses were conducted in three models as follows: unadjusted (Model 0), adjusted for sex, age and BMI (Model 1), and additionally adjusted for smoking and drinking status (Model 2). A *p*-value < 0.05 was considered significant.

## 3. Results

### 3.1. General Characteristics of Participants According to Metabolic Syndrome

As shown in Table 1, study participants were divided into two groups: non-MetS (*n* = 1699) and MetS (*n* = 299). The MetS group had a higher proportion of females, contained older and heavier participants, consumed less alcohol, and smoked fewer cigarettes than the non-MetS group. Therefore, these parameters were controlled for further analysis. As expected, the MetS group exhibited significantly larger WCs, higher BPs, higher TG and FBG levels, and lower HDLC levels than the non-MetS group. In addition, serum levels of TC, LDLC, AST, and ALT were also higher in the MetS group than in the non-MetS group. The statistical significances were maintained after the adjustment.

### 3.2. Circulating Amino Acid Metabolite Ratios Associated with Arginase and Nitric Oxide Synthase Activities According to Metabolic Syndrome Risk Status

**Figure 2:**
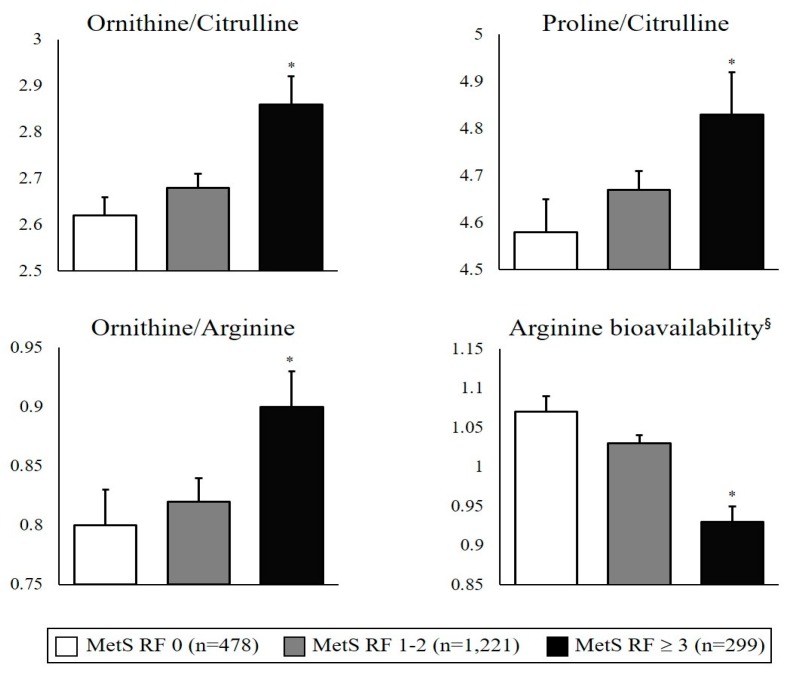
Association between circulating amino acids metabolites and MetS risk status. Data are presented as mean ± standard error; tested after log-transformed; tested by general linear model (GLM) with Bonferroni’s multiple comparisons test (adjustment for age, sex, BMI, cigarette smoking and alcohol consumption); MetS RF: MetS risk factor. * represents significant differences in the values between Mets RF ≥ 3 group and MetS RF 0/MetS RF 1–2 groups; ^§^ Arginine bioavailability is defined as Arginine/(Ornithine + Citrulline).

Ratios of circulating amino acid metabolites, namely ornithine, citrulline, proline, and arginine, associated with arginase and NOS activities and arginine bioavailability, were individually measured, and subsequently, ratios of these metabolites were calculated. The ornithine to citrulline (ornithine/citrulline) and proline to citrulline (proline/citrulline) ratios indirectly indicated the arginase/NOS activity ratio, whereas the ornithine/arginine ratio directly indicated arginase activity. As shown in Figure 2, with the increase in the number of MetS risk factors, ornithine/citrulline, proline/citrulline, and ornithine/arginine ratios also significantly increased (MetS risk factor = 0, *n* = 478; MetS risk factor = 1–2, *n* = 1221; MetS risk factor ≥3, *n* = 299). On the other hand, arginine bioavailability, defined as arginine/(ornithine + citrulline), significantly decreased with the increase in the number of MetS risk factors.

### 3.3. Correlations between Amino Acid Metabolites and Metabolic Syndrome Risk-Related Parameters

**Figure 3:**
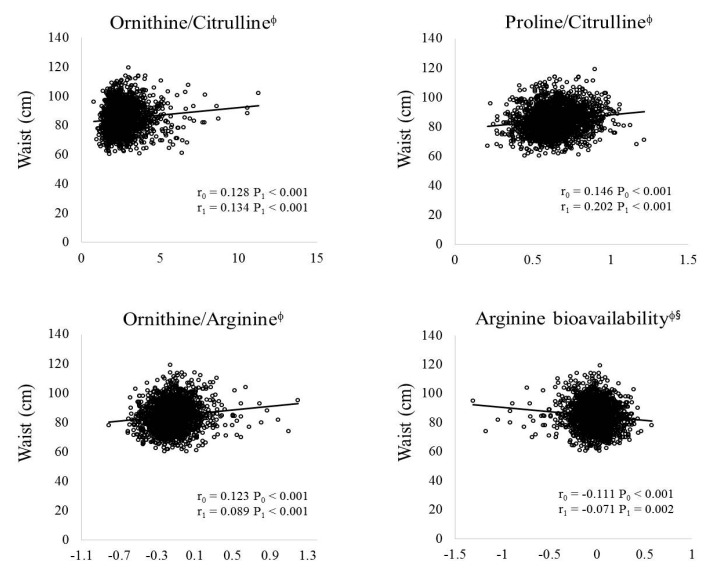
Relationship between circulating amino acids metabolites and waist circumference. Data are represented as mean ± standard error; *r*—correlation co-efficient, *p*: *p*-value. Data were tested by Pearson (*r*_0_, *P*_0_, unadjusted) and Partial (*r*_1_, *P*_1_: adjusted for age, sex, BMI, cigarette smoking and alcohol consumption) correlation analyses. ^φ^ tested after log-transformed; ^§^ Arginine bioavailability = Arginine/(Ornithine + Citrulline).

Pearson (*r*_0_, *P*_0_) and partial (*r*_1_, *P*_1_) correlation analyses were performed to identify the relationship between the MetS risk-related metabolic parameters and the ratios of amino acid metabolites. As shown in Figure 3, WCs were positively correlated with the ornithine/citrulline ratio (*r*_0_ = 0.128, *P*_0_ < 0.001; *r*_1_ = 0.134, *P*_1_ < 0.001), proline/citrulline ratio (*r*_0_ = 0.146, *P*_0_ < 0.001; *r*_1_ = 0.202, *P*_1_ < 0.001), and ornithine/arginine ratio (*r*_0_ = 0.123, *P*_0_ < 0.001; *r*_1_ = 0.089, *P*_1_ < 0.001) but were negatively correlated with arginine bioavailability (*r*_0_ = −0.111, *P*_0_ < 0.001; *r*_1_ = −0.071, *P*_1_ = 0.002) before and after adjustments for age, sex, BMI, cigarette smoking, and alcohol consumption. In addition, after the adjustment, serum levels of TG (*r*_1_ = 0.062, *P*_1_ < 0.05), FBG (*r*_1_ = 0.051, *p* < 0.05), AST (*r*_1_ = 0.114, *P*_1_ < 0.05), and ALT (*r*_1_ = 0.097, *P*_1_ < 0.05) were positively correlated but those of HDLC (*r*_1_ = −0.051, *P*_1_ < 0.05) were negatively correlated with the ornithine/citrulline ratio (Table 2). DBPs (*r* = 0.035, *p* < 0.05), TC levels (*r*_1_ = 0.028, *P*_1_ < 0.05), and ALT levels (*r*_1_ = 0.079, *P*_1_ < 0.05) were positively correlated but AST levels (*r*_1_ = −0.005, *P*_1_ < 0.05) were negatively correlated with the proline/citrulline ratio. Serum levels of TG (*r*_1_ = 0.027, *P*_1_ < 0.05) and ALT (*r*_1_ = 0.054, *P*_1_ < 0.05) were positively correlated but those of HDLC (*r*_1_ = −0.088, *P*_1_ < 0.05), and FBG (*r*_1_ = −0.002, *P*_1_ < 0.05) were negatively correlated with the ornithine/arginine ratio. On the other hand, arginine bioavailability was positively correlated with serum levels of HDLC (*r*_1_ = 0.110, *P*_1_ < 0.05), and TC (*r*_1_ = 0.074, *P*_1_ < 0.05) but negatively correlated with DBPs (*r*_1_ = −0.051, *P*_1_ = 0.05) and AST levels (*r*_1_ = −0.107, *P*_1_ < 0.05) (Table 2).

### 3.4. Association between Circulating Amino Acid Metabolite Ratios and Metabolic Syndrome Risk

Associations between amino acid metabolite ratios and MetS risk were evaluated by comparing ORs (95% CIs), which were calculated using a logistic regression model with adjustments for confounding factors (namely age, sex, BMI, cigarette smoking, and alcohol consumption) (Table 3). Study population was divided into quartile groups according to each amino acid metabolite ratio. The highest quartile group of the ornithine/citrulline ratio demonstrated higher MetS risk than the lowest quartile group before and after the adjustment (OR_0_: 1.98, 95% CI: 1.39–2.82, *P*_0_ < 0.001; OR_1_: 1.75, 95% CI: 1.18–2.59, *P*_1_ = 0.005; OR_2_: 1.71, 95% CI: 1.15–2.54, *P*_2_ = 0.008). In addition, the highest quartile group of the ornithine/arginine ratio demonstrated higher MetS risk than the lowest quartile group before and after the adjustment (OR_0_: 2.89, 95% CI: 1.98–4.22, *P*_0_ < 0.001; OR_1_: 2.58, 95% CI: 1.70–3.90, *P*_1_ < 0.001; OR_2_: 2.57, 95% CI: 1.69–3.90, *P*_2_ < 0.001). However, the proline/citrulline ratio was not significantly associated with MetS, whereas arginine bioavailability was negatively associated with MetS (OR_0_: 0.38, 95% CI: 0.26–0.55, *P*_0_ < 0.001; OR_1_: 0.40, 95% CI: 0.26–0.60, *P*_1_ < 0.001; OR_2_: 0.40, 95% CI: 0.26–0.61, *P*_2_ < 0.001). Next, multiple regression analysis was performed to identify the association of contributing factors, amino acid metabolite ratios, and arginine bioavailability with MetS risk. ORs for the association of the ornithine/citrulline ratio and arginine bioavailability with MetS were 1.33 (95% CI: 0.87–2.05, *p* = 0.188) and 0.45 (95% CI: 0.29–0.69, *p* < 0.001), respectively, after the adjustment. ORs for the association of the proline/citrulline ratio and arginine bioavailability with MetS were 1.48 (95% CI: 0.97–2.25, *p* = 0.070) and 0.38 (95% CI: 0.25–0.58, *p* < 0.001), respectively, after the adjustment. ORs for the association of the ornithine/arginine ratio and arginine bioavailability with MetS were 2.62 (95% CI: 0.92–7.44), *p* = 0.070) and 0.97 (95% CI: 0.34–2.78, *p* = 0.961), respectively, after the adjustment.

## 4. Discussion

This study demonstrated that alterations in circulating amino acid metabolite ratios, which are indicative of arginase and NOS activities, and arginine bioavailability were significantly associated with MetS risk: people with high ratios of circulating metabolites (particularly, those of ornithine/citrulline and ornithine/arginine) and low levels of arginine bioavailability demonstrated high MetS risk. These results suggested that circulating amino acid metabolite ratios, which are indicative of arginase activity and arginine bioavailability can potentially serve as biomarkers for assessing MetS risk.

Accumulating evidence has shown strong association of a group of metabolic disorders, such as obesity, insulin resistance, hyperglycemia, diabetes, CVD, and atherosclerosis [26,27], with arginase and NOS pathways of l-arginine substrate [10,11,12,28,29,30,31,32,33,34,35,36,37,38,39]. Arginase isoforms, which are involved in the urea cycle and are expressed in the hepatic, vascular smooth muscle, and endothelial cells, are responsible for converting l-arginine to urea and l-ornithine, and l-ornithine is further metabolized to proline by ornithine aminotransferase [18,40]. Arginases are activated by inflammatory factors and atherothrombosis mediators, including oxidized low-density lipoprotein [41] and thrombin [42], thereby inhibiting NO-mediated endothelial relaxation. The upregulated arginase activity also contributes to the lack of l-arginine for NOS pathway, which decreases NO bioavailability and leads to various metabolic complications, such as diabetes, inflammation, and cardiovascular disorders, including endothelial dysfunction, high BP, diabetic vascular disease, and atherosclerosis [20,37,43,44,45]. Recent studies have indicated that increased NO production can result from arginase inhibition or l-arginine supplementation, which reduces the arginase activity [10,11,12,13,14,15,16,17]. These results have demonstrated the importance of arginase inhibition in endothelial dysfunction, which occurs in several pathological states, such as atherosclerotic and vascular disease. In addition, arginase inhibition plays an important role in restoring metabolic disorders, such as T2DM [10,37], endothelial dysfunction in adjuvant arthritis [34], hypertension [32] and atherosclerosis [46]. In our previous studies, arginase inhibition not only ameliorated body fat, hepatic lipid abnormalities, and adipose tissue inflammation but also restored endothelial dysfunction in obesity-induced animals, which was mediated by increased NO production [11,12,43]. Emerging evidence has indicated that NOS-synthesized NO plays a key role in regulating energy metabolism and pathogenesis of metabolic abnormalities, indicating that NO output may be beneficial for preventing and treating obesity and insulin resistance [47]. NO bioavailability is reduced in diet-induced obese animals [48,49] and in overweight humans with insulin resistance [50]. Furthermore, increased endothelial NOS (eNOS) activity prevents the obesogenic effects of a high-fat diet, which indicates the anti-obesity effect of eNOS in regulating lipid metabolism [51]. Thus, enhanced physiological levels of NO can ameliorate all disorders in obese conditions and reduce the arginase activity [52].

To date, accumulating evidence has shown the important implications of arginase activity on MetS but has demonstrated limited information about the effect of altered amino acid metabolite ratios on arginase activity in the presence of metabolic abnormalities. Our previous study has demonstrated that arginase gene expression is significantly upregulated in overweight people and that arginase mRNA levels are closely associated with phenotype biomarkers for obesity, disturbed lipid profiles, and endothelial dysfunction [28]. In the present study, we analyzed arginine, a substrate of arginase and NOS, and the secondary products produced by these two enzymes (ornithine, citrulline, and proline) and found a significant correlation between these amino acid metabolite ratios and MetS risk-related parameters. In this study, arginine and its catabolic metabolites including ornithine, proline, and citrulline were used for the indicator of arginase and NOS since there is with no net loss of these metabolites during urea synthesis. Among these parameters, WC was significantly correlated with all amino acid metabolite ratios; it was positively correlated with ornithine/citrulline, proline/citrulline, and ornithine/arginine ratios but negatively correlated with arginine bioavailability. As stated above, ornithine/citrulline and proline/citrulline ratios indirectly indicated the arginase/NOS activity ratio, whereas the ornithine/arginine ratio directly indicated arginase activity. In this study, ornithine/citrulline, proline/citrulline, and ornithine/arginine ratios were significantly increased with the increase in the number of MetS risk factors. The increased ornithine/citrulline and proline/citrulline ratios indicated that arginase activity was higher than NOS activity. The increased ornithine/arginine ratio implied that arginase activity was upregulated but NOS activity was downregulated. On the other hand, arginine bioavailability significantly decreased with the increase in the number of MetS risk factors, which suggested that the levels of arginine available as a substrate for arginase and NOS were low in the MetS status. In addition, multiple regression analyses revealed that arginine bioavailability was more independent of risk factors than arginase and NOS activities. These data indicated that circulating amino acid metabolite ratios reflect arginase and NOS activities and may explain the potential mechanism of arginase and NOS pathways in metabolic disorders. On the other hand, it should be noted that the ratio of proline to citrulline was not associated with the ORs for MetS, possibly due to the fact that proline is not a direct metabolite of arginase.

This study has a limitation. Measurements of circulating amino acids were not repeated. This is a potential limitation because circulating enzymes in blood can occasionally change; however, because the data were collected from a relatively large population, such changes and, consequently, the limitation can be overlooked. Despite this limitation, the present study demonstrated a strong relationship between circulating amino acids and MetS-related risk parameters, suggesting that alterations in circulating amino acid metabolite ratios, which are associated with arginase and NOS activities, and arginine bioavailability are potential indicators for assessing MetS risk. These alterations may also indicate important information for deciphering the pathogenesis of disturbances in amino acid metabolism related to the arginase activity.

## Figures and Tables

**Table 1:** Basic population characteristics according to MetS status.

Total (*n* = 1998)	Non-MetS (*n* = 1699)	MetS (*n* = 299)	*P*_0_	*P*_1_	*P*_2_
Age (years)	56.02 ± 0.22	59.92 ± 0.50	<0.001	-	-
Male (%)	48.73	28.43	<0.001	-	-
Body weight (kg) ^φ^	61.23 ± 0.25	65.88 ± 0.59	<0.001	-	-
BMI (kg/m^2^) ^φ^	23.89 ± 0.07	26.82 ± 0.17	<0.001	-	-
Physical activity (%)					
Low	14.83	11.41	0.292	0.296	0.454
Moderate	36.35	37.25			
High	48.82	51.34			
Drinking status (%)					
Never	47.00	55.85	0.018	<0.001	-
Former	4.71	3.68			
Current	48.29	40.47			
Smoking status (%)					
Never	61.13	74.92	<0.001	<0.001	-
Former	18.14	8.70			
Current	20.73	16.39			
MetS risk factors					
WC (cm)	83.13 ± 0.21	93.44 ± 0.45	<0.001	-	<0.001
TG (mg/dL) ^φ^	121.63 ± 1.90	233.57 ± 11.31	<0.001	<0.001	<0.001
HDLC (mg/dL) ^φ^	45.55 ± 0.25	37.85 ± 0.40	<0.001	<0.001	<0.001
FBG (mg/dL) ^φ^	89.85 ± 0.25	95.97 ± 0.68	<0.001	<0.001	<0.001
SBP (mmHg)	114.20 ± 0.37	129.14 ± 0.90	<0.001	<0.001	<0.001
DBP (mmHg)	74.98 ± 0.23	84.19 ± 0.57	<0.001	<0.001	<0.001
Biochemical parameters					
TC (mg/dL)	191.08 ± 0.85	202.56 ± 2.24	<0.001	<0.001	<0.001
LDLC (mg/dL)	121.43 ± 0.74	125.34 ± 2.08	0.054	<0.001	<0.001
AST (IU/L) ^φ^	25.21 ± 0.27	27.74 ± 2.20	0.271	<0.001	<0.001
ALT (IU/L) ^φ^	22.49 ± 0.41	24.97 ± 1.06	0.003	<0.001	<0.001

Data are presented as mean ± standard error or percentage (%). ^φ^ tested after log-transformed; tested by *t*-test (unadjusted), general linear model (GLM) with Bonferroni’s multiple comparisons test (adjusted), or chi square test; *P*_0_: unadjusted *p*-value; *P*_1_: *p*-value after adjusting for age, sex and BMI. *P*_2_: *p*-value after adjusting for age, sex, BMI, cigarette smoking and alcohol consumption; ALT: alanine aminotransferase; AST: aspartate aminotransferase; DBP: diastolic BP; SBP: systolic BP; FBG: fasting blood glucose; HDLC: high density lipoprotein cholesterol; LDLC: low-density lipoprotein cholesterol; MetS: metabolic syndrome; TC, total cholesterol; TG, triglyceride; WC, waist circumference.

**Table 2:** Correlation between circulating amino acid metabolites, MetS risk factors, and related biochemical parameters.

Total (*n* = 1998)	ORN/CIT	PRO/CIT	ORN/ARG	Arginine Bioavailability ^§^
TG (mg/dL) ^φ^	0.062 *	0.149	0.027 *	−0.026
HDLC (mg/dL) ^φ^	−0.051 *	−0.056	−0.088 *	0.110 *
FBG (mg/dL) ^φ^	0.051 *	0.134	−0.002 *	0.021
SBP (mmHg)	0.020	0.038	0.021	−0.029
DBP (mmHg)	0.041	0.035 *	0.054	−0.051 *
TC (mg/dL)	0.010	0.028 *	−0.048	0.074 *
LDLC (mg/dL)	−0.017	−0.022	−0.043	0.044
AST (IU/L) ^φ^	0.114 *	−0.005 *	0.170	−0.107 *
ALT (IU/L) ^φ^	0.097 *	0.079 *	0.054 *	−0.025

Correlation coefficient, tested by partial correlation analysis with adjustment for age, sex, BMI, and smoking status and alcohol consumption. ^φ^ tested after log-transformed; * *p*-value < 0.05; ^§^ Arginine bioavailability = Arginine/(Ornithine + Citrulline); ALT, alanine aminotransferase; AST, aspartate aminotransferase; DBP, diastolic blood pressure; FBG, fasting blood glucose; HDLC, high-density lipoprotein cholesterol; LDLC, low-density lipoprotein cholesterol; SBP, systolic blood pressure; TC, total cholesterol; TG, triglyceride.

**Table 3:** Risk of MetS associated with circulating amino acid metabolites.

Total (*n* = 1998)	OR_0_ (95% CI) *p*-Value	OR_1_ (95% CI) *p*-Value	OR_2_ (95% CI) *p*-Value
ORN/CIT ^φ^	1.98 (1.39, 2.82) <0.001	1.75 (1.18, 2.59) 0.005	1.71 (1.15, 2.54) 0.008
PRO/CIT ^φ^	1.29 (0.91, 1.85) 0.154	1.33 (0.88, 2.01) 0.174	1.30 (0.86, 1.97) 0.215
ORN/ARG ^φ^	2.89 (1.98, 4.22) <0.001	2.58 (1.70, 3.90) <0.001	2.57 (1.69, 3.90) <0.001
Arginine bioavailability ^φ,§^	0.38 (0.26, 0.55) <0.001	0.40 (0.26, 0.60) <0.001	0.40 (0.26, 0.61) <0.001

OR: odds ratio, CI: confidence interval, ^φ^ tested after log-transformed; The association was calculated using the OR (95% CIs) of a logistic regression model (OR_0_: unadjusted, OR_1_: adjusted for age, sex, and BMI, OR_2_ adjusted for age, sex, BMI and smoking status and alcohol consumption). ^§^ Arginine bioavailability = Arginine/(Ornithine + Citrulline); ARG, Arginine; CIT, Citrulline; ORN, Ornithine; PRO, Proline.
